# Mechano-Triboelectric Analysis of Surface Charge Generation on Replica-Molded Elastomeric Nanodomes

**DOI:** 10.3390/mi12121460

**Published:** 2021-11-27

**Authors:** Myung Gi Ji, Mohammed Bazroun, In Ho Cho, W. Dennis Slafer, Rana Biswas, Jaeyoun Kim

**Affiliations:** 1 Department of Electrical and Computer Engineering, Iowa State University, Ames, IA 50011, USA; mji90@iastate.edu; 2 Microelectronics Research Center, Iowa State University, Ames, IA 50011, USA; 3 Department of Civil, Construction, and Environmental Engineering, Iowa State University, Ames, IA 50011, USA; mbazroun@iastate.edu (M.B.); icho@iastate.edu (I.H.C.); 4 MicroContinuum Inc., 200 Dexter Avenue, Suite 180, Watertown, MA 02472, USA; harveyfletcher@yandex.com; 5 Department of Physics and Astronomy, Iowa State University, Ames, IA 50011, USA; 6 Ames Laboratory, Iowa State University, Ames, IA 50011, USA

**Keywords:** triboelectricity, contact electrification, nanopatterned tribocharge, mechano-triboelectric charging model, Kelvin probe force microscopy

## Abstract

Replica molding-based triboelectrification has emerged as a new and facile technique to generate nanopatterned tribocharge on elastomer surfaces. The “mechano-triboelectric charging model” has been developed to explain the mechanism of the charge formation and patterning process. However, this model has not been validated to cover the full variety of nanotexture shapes. Moreover, the experimental estimation of the tribocharge’s surface density is still challenging due to the thick and insulating nature of the elastomeric substrate. In this work, we perform experiments in combination with numerical analysis to complete the mechano-triboelectrification charging model. By utilizing Kelvin probe force microscopy (KPFM) and finite element analysis, we reveal that the mechano-triboelectric charging model works for replica molding of both recessed and protruding nanotextures. In addition, by combining KPFM with numerical electrostatic modeling, we improve the accuracy of the surface charge density estimation and cross-calibrate the result against that of electrostatic force microscopy. Overall, the regions which underwent strong interfacial friction during the replica molding exhibited high surface potential and charge density, while those suffering from weak interfacial friction exhibited low values on both. These multi-physical approaches provide useful and important tools for comprehensive analysis of triboelectrification and generation of nanopatterned tribocharge. The results will widen our fundamental understanding of nanoscale triboelectricity and advance the nanopatterned charge generation process for future applications.

## 1. Introduction

There has been an increasing level of interest in generating electric charges in a nanoscale patterned format for applications such as nano-xerography [1,2] and ultra-high-density data storage [3,4]. Conventional methods for nanopatterned charge generation include focused irradiation of pulsed lasers [5,6], charge injection from nanopatterned electrodes [7,8], and chemical deposition through nanoscale stencils [9]. However, those approaches often necessitate complex cleanroom processes and costly tools.

**Figure 1:**
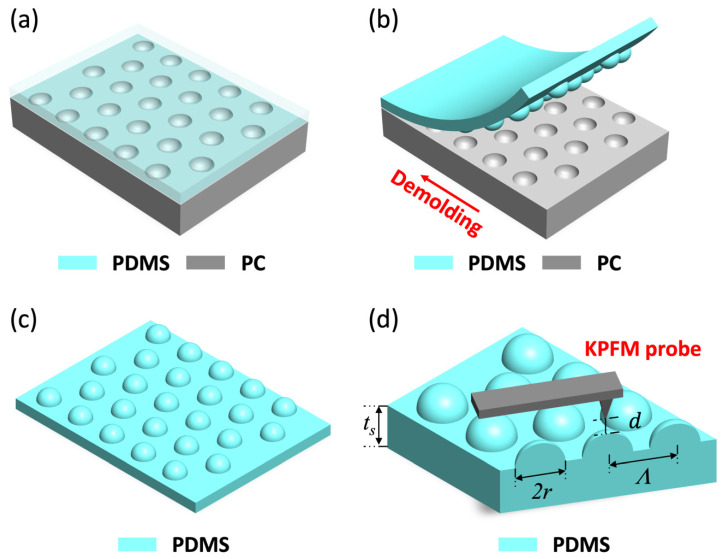
Replica molding-based tribocharge nanopatterning process. (**a**) A PC nanocup master mold is replicated with PDMS. (**b**) Demolding process of the PDMS replica. The red arrow indicates the direction of demolding. (**c**) The resulting PDMS nanodome with its diameter 2*r* ~ 500 nm, and pitch Λ *~* 750 nm, respectively. (**d**) KPFM and EFM scanning of the PDMS nanodome to map the distribution of tribocharge (*t*_s_: sample thickness, *d*: tip lift-height).

Recently, we reported a new method for generating nanopatterned electric charges [10,11,12,13] by exploiting the technique of elastomeric replica molding [14]. Figure 1 shows the process. First, liquid-phase poly(dimethylsiloxane) (PDMS) was poured over a nanotextured polymer master mold (Figure 1a). Upon its complete curing, the PDMS replica was peeled off from the master mold (Figure 1b,c). Due to the frictional interaction between the master mold and PDMS replica during the peel-off action, their surfaces become triboelectrically charged, acquiring the tribocharge. By jointly utilizing atomic force microscopy (AFM) and Kelvin probe force microscopy (KPFM) for surface topography and potential [11,12,15] respectively (Figure 1d), we revealed that the spatial distribution of the tribocharge’s density exhibited nanoscale patterns that are closely correlated with the shape of the master mold’s nanotexture, rather than being random or uniform. For example, on PDMS nanocups replicated from polycarbonate (PC) nanodomes, we observed that the tribocharges were concentrated mainly around the rims of the nanocups, forming nanoscale “ring charges” [10]. The polarity of the tribocharge was determined primarily by the master mold’s material composition [16]. This new technique is simple, cost-effective, and inherently compatible with PDMS, which is gaining importance as the platform material for energy harvesting [4,5,6,7,8,9,17,18] and flexible electronics [19,20,21].

To diversify the tribocharge’s distribution pattern attainable with this technique, it was imperative to elucidate how the mechanics of the peel-off action are related to the spatial density distribution of the tribocharge resulting from it. To that end, we used a two-way approach in which we first simulated the peel-off action using the finite element method (FEM), then obtained the spatial distribution patterns of various mechanical parameters related to the peel-off action, and finally compared them with that of the tribocharge. Through the study, we showed that the tribocharge’s distribution pattern was most closely related to and, hence, can be best explained and predicted by the lateral sliding distance, which represents the cumulative lateral friction. The resulting “mechano-triboelectric charging model” of the nanopatterned tribocharge generation process successfully explained the appearance of the complex partial eclipse-shaped tribocharge nanopatterns, rather than the rings reported in [10], when the master mold’s material was changed from PC to polyethylene terephthalate (PET) [12].

However, the mechano-triboelectric charging model exhibited two deficiencies. First, it has been tested only with master molds with protruding nanotextures, such as the PC and PET nanodome arrays used in [10,12]. Whether the model can be extended to the triboelectrification by the replication of recessed nanotextures, such as the PC nanocups in Figure 1a, has not been tested experimentally. Second, the process of the replica molding-based tribocharging inevitably puts the charge on the PDMS replica. The thickness of the PDMS substrate (*t*_s_ in Figure 1d) often reaches hundreds of microns, separating the surface charge and the electrical ground point by the same distance. It can greatly obscure the relation between the surface potential measured by KPFM and the true characteristics of the surface charge, such as its density, and harms the validity of the mechano-triboelectric charging model.

In this work, we carry out a multi-physical investigation to demonstrate that our mechano-triboelectric charging model is also applicable to the replication of recessed nanotextures using master molds textured with nanocup arrays, as shown in Figure 1. To improve the accuracy of KPFM in our replica molding-based tribocharging setup with a “thick dielectric substrate”, we reinforce KPFM with numerical electrostatic simulations [22,23,24] and cross-check the results with those obtained with electrostatic force microscopy (EFM) [12,15]. We anticipate the results will widen our understanding of nanoscale triboelectricity and advance the technology of nanopatterned charge generation.

## 2. Materials and Methods

### 2.1. Tribocharging and Analysis

**Figure 2:**
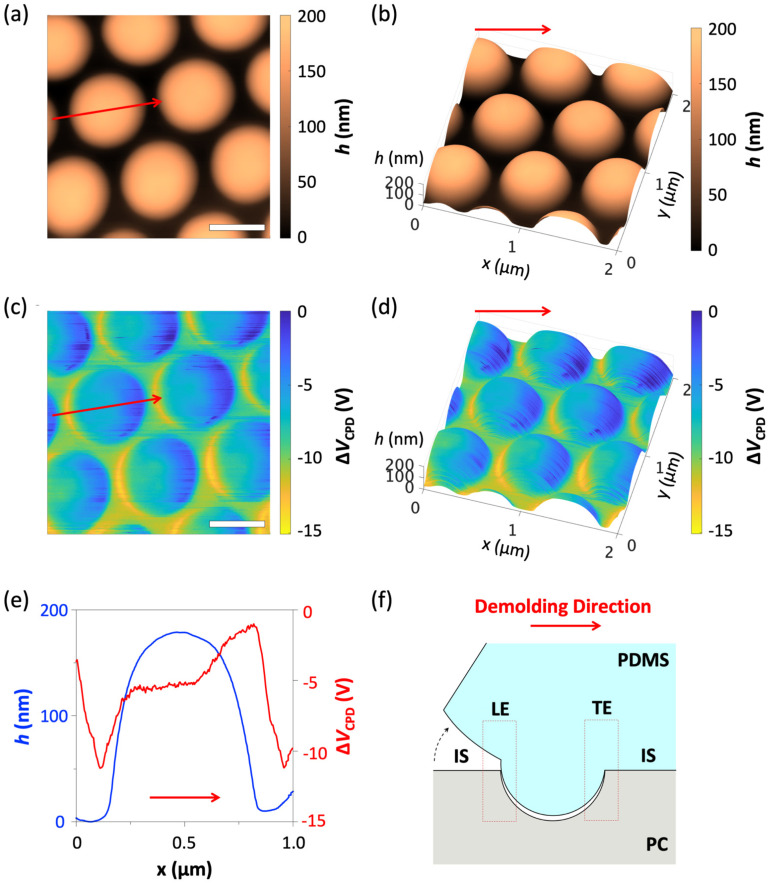
(**a**) AFM scans of the PDMS nanodome replica with aspect ratio (AR = 0.76). The red arrow specifies the demolding direction (scale bar: 500 nm). (**b**) Bird’s eye views of the AFM scans in (**a**). (**c**) The corresponding surface potential map obtained with KPFM (scale bar: 500 nm). (**d**) Bird’s eye views of the KPFM scans in (**c**). (**e**) The height (blue solid) and potential (red solid) profiles are superimposed for facile comparison. (**f**) A schematic diagram of the basic mechanics of the peel-off action (LE: leading edge, TE: trailing edge, IS: interstitial region).

We first carried out the replica molding-based tribocharging process by taking the steps described in Figure 1. As the master mold for this work, we adopted polycarbonate (PC) nanocup arrays (MicroContinuum Inc., Watertown, MA, USA). The nanocups measure 250 nm in radius and form a 750 nm pitch (Λ) triangular lattice. We then prepared liquid phase PDMS (Sylgard 184, Dow Corning) mixed with a curing agent at a 10:1 wt.% ratio and poured it over the PC master mold (Figure 1a). The prepared sample was cured at −65 °C for 24 h. Then, the completely cured PDMS replica was demolded from the PC nanocup array by manual peel-off, as shown in Figure 1b. The direction of the peel-off action, often referred to as the demolding direction, is indicated by an arrow in Figure 1b. The resulting PDMS replica took the form of a triangular nanodome array (Figure 1c). Figure 2a,c show the AFM scans of the PDMS nanodome replica. The radius, *r*, and pitch, Λ, were 250 and 750 nm respectively, faithfully matching those of the PC nanocup array. The height of the nanodome was measured to be 187.5 ± 11.4 nm (s.d.), setting the aspect ratio AR = *h*/*r* at 0.76.

Subsequently, the surface of the PDMS replica was characterized by KPFM and EFM. The two techniques are ideal for relating the topography of the surface and the density of the tribocharge formed on it because they acquire topographic and electric information from the surface simultaneously. In our work, the PDMS replica was first cut into a 1 × 1 cm square block and placed in our AFM (Multimode, Bruker, Billerica, MA, USA). The KPFM and EFM measurements were performed using a conductive tip (SCM-PtSi, Bruker, Billerica, MA, USA, *k* = 2.8 N/m, *f*_o_ = 75 kHz, Bruker). KPFM firstly scans the surface topography in the tapping mode and then lifts the tip at a fixed height to obtain the surface potential data. Herein, the lift height, *d*, was set to 50 nm. The scanning rate and area were set to 0.5 Hz and 2 × 2 μm, respectively.

Figure 2b,d show the surface potential obtained with KPFM from the replica in the top and bird’s eye views, respectively. Since the thick slab of highly insulating PDMS caused an unknown level of potential drop, it was difficult to find the absolute surface potential measured from the ground point, i.e., the bottom electrode. Since we already know that PDMS replication of PC generates negative charges on PDMS from our previous work [10], we set the highest potential level to zero and plotted the relative surface potential, ΔV_CPD_, with respect to it. The surface potential clearly exhibited a non-uniform distribution pattern. To relate the surface topography and potential more facilely, we have extracted the two quantities along the red arrows in Figure 2a,b and plotted them in superposition in Figure 2e as blue and red curves, respectively.

We found that the relation between the surface topography and the surface potential level, which must be proportional to the surface charge density, agreed well with the basic mechanics of the peel-off action, qualitatively. During the peel-off action described schematically in Figure 2f, the leading edge (LE) and the trailing edge (TE) should undergo the maximum and minimum levels of interfacial friction, respectively. Indeed, the LE of the nanodomes which suffered from the highest level of friction exhibited the largest change in potential (approximately −10 V). The surface potential in the TE of the nanodome showed the smallest change level because it had suffered the lowest level of friction. In the flat interstitial (IS) region between the nanodomes, the surface potential stayed at the mid-level (approximately −5 V). Near the top of the nanodome, the surface potential returned to the IS level (approximately −5 V). This behavior, which conforms well with the mechano-triboelectric charging model, was observed on most PDMS nanocups in the probing area.

### 2.2. Mechano-Triboelectric Charging Model

To further verify the mechano-triboelectric charging model of the surface charge distribution, we carried out a nonlinear finite element analysis (FEA) of the cohesive demolding process and compared the results with the experimental measurements. Due to the inherently curved nature of the nanocups and nanodomes, the peel-off action occurred in a mixed mode comprising pure crack opening and sliding modes simultaneously. Therefore, the frictional stress was assessed through the adoption of the mixed mode cohesive zone model (CZM) in the presence of nonlinearities in both the material characteristics and the geometry.

**Figure 3:**
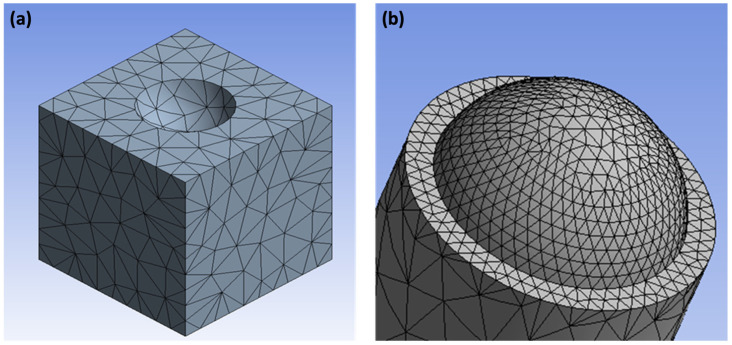
The setup for the finite element analysis (FEA) of the demolding action between (**a**) a polycarbonate (PC) nanocup master mold and (**b**) a poly(dimethylsiloxane) (PDMS) nanodome replica.

All computational simulations were conducted on ANSYS (Ansys, Release 2020 R1, Canonsburg, PA, USA) using 3D geometry. Due to the length-scale limitation of the continuum FEA in ANSYS, the simulations were carried out at micrometer length-scales, while preserving all the geometric features. In the model setting, two faces of the PDMS and PC were bonded and contacted; hence, the mesh size of the contacted faces has been controlled to be 30 μm for each element size, while the other faces have a medium smoothing mesh (Figure 3). In addition, the debonding directional vector was set to be −1 and −200 in x- and y-direction, respectively.

The material and failure characteristics of the interface elements were obtained from the literature. Specifically, Young’s modulus and Poisson’s ratio were set to 1.0 MPa and 0.45, respectively. The CZM was defined with 15 KPa for the normal and shear strengths and 330 µm for the separation limit. We assumed a clear interfacial failure without any fracture of PDMS fibrils, which agrees well with the experimental observations. Since we have already identified the lateral sliding distance, *L*_s_, as the governing factor of the mechano-triboelectric surface charge generation in our previous work [12], we adopted it again in this work for explaining and interpreting the measured charge distribution.

**Figure 4:**
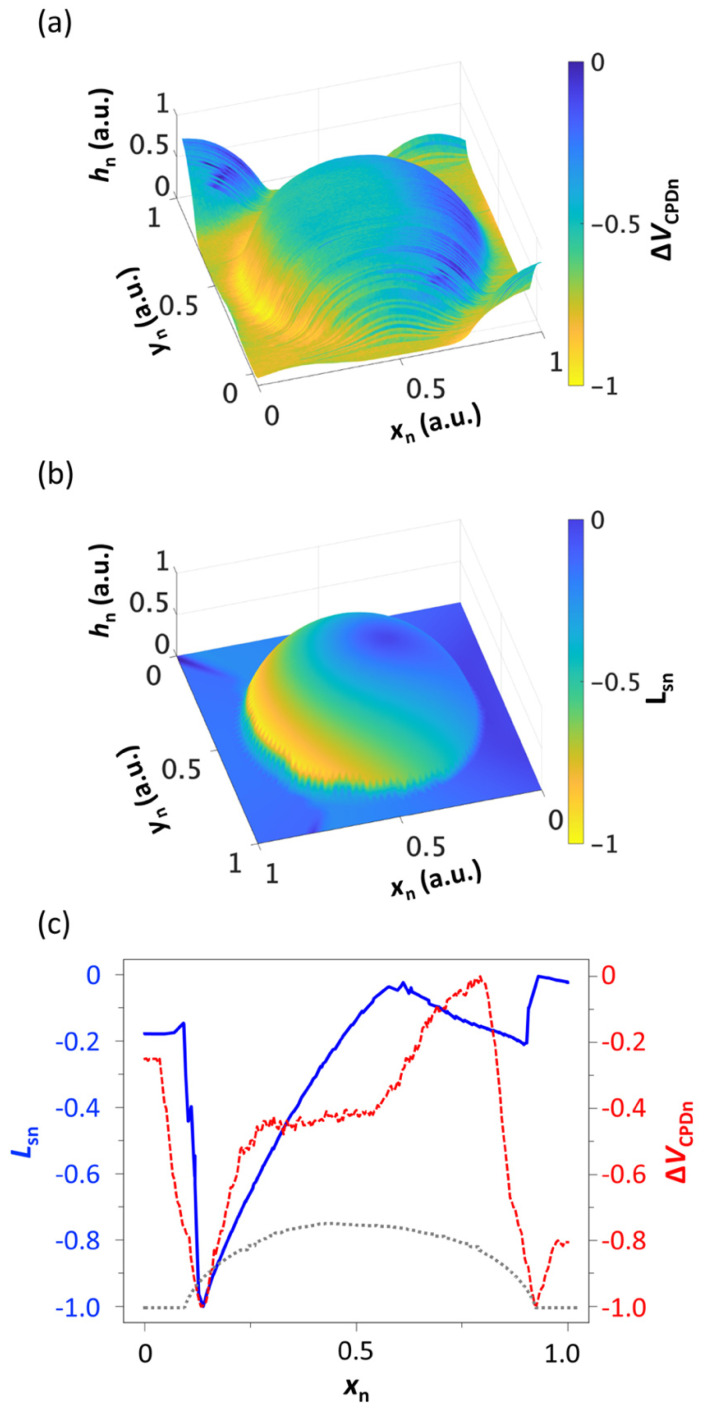
(**a**) A bird’s eye view of the surface potential distribution over a single PDMS nanodome. (**b**) The corresponding distribution of the computed lateral sliding distance, *L*_sn_. Both the surface potential and the lateral sliding distance are normalized to their maximum values to facilitate the comparison. (**c**) Δ*V*_CPDn_ (red dashed) and *L*_sn_ (blue solid) profiles along the center line of the nanodome are superimposed for facile comparison. The surface topography is also added (gray dashed).

For facile comparison, we plotted the surface potential distribution, ∆*V*_CPDn_, of a single nanodome in Figure 4a and the corresponding *L*_sn_ in Figure 4b. For facile comparison, both quantities were normalized to their maximum values. The distribution patterns of the two normalized quantities exhibit a high level of similarity, with their maximum and minimum areas located at the LE and TE sides respectively, re-confirming that the lateral sliding distance is the main governing factor of the replica molding-based tribocharging process. The mechanics of the demolding process, shown in Figure 2f, also indicate that the maximum sliding between the interfaces occurs at the LE and the minimum at the TE.

For a more quantitative comparison, the two quantities, *L*_sn_ and ∆*V*_CPDn_, were sampled along the center line of the nanodome and plotted in superposition in Figure 4c. Their agreement is good in both the LE and TE regions, which correspond to the areas of the highest and lowest level of interfacial friction, respectively. The agreement is, however, weaker in other areas. The biggest discrepancy occurs near the top region of the nanodome. We ascribe the discrepancy to the imperfection in simulating mechanical interactions between soft, highly deformable structures. Of strongest impact may be the fact that the FEA model consists of only one nanodome, while the experimental structure comprised multiple nanodomes arranged in a triangular lattice pattern. We anticipate that more accurate and realistic modeling will enhance the level of agreement between the two quantities.

As an additional validation of the mechano-triboelectric model, we have also investigated another structure, a 3D pyramidal mold, using FEA. The modeling results can be found in the Supplementary Materials, Figure S1.

### 2.3. Surface Charge Density Estimation

With the mechano-triboelectric charging model re-affirmed for the production of tribocharges through replication of recessed nanotextures, we proceeded to quantify the density of the surface charge. So far, we have quantified the surface charge density using two different methods. For a highly symmetric charge distribution, such as the ring charge distribution in [10], we took a semi-analytical approach, in which the charge distribution was modeled as a uniform or non-uniform assembly of point charges. We first assigned charge values to each point charge, analytically computed the total electric potential due to all point charges within the probing area of KPFM, and then compared the resulting potential with that measured by KPFM. These steps were iterated until the computation and KPFM results agreed with each other. However, the semi-analytical approach is difficult to apply to asymmetric charge distributions, such as the partial eclipse pattern observed in [12], because computing its total electric potential often escapes the realm of analytical integration, necessitating complex numerical integrations. Moreover, particularly for the tribocharges generated by the replica molding technique, there exists one more challenge: the thickness of the PDMS replica (*t*_s_ in Figure 1d). In typical replica molding using PDMS, it is common to set *t*_s_ in the range of hundreds of microns to avoid mechanical failures during the experimental peel-off action. In the KPFM setup, this thickness separates the surface charge (on the upper surface) from the bottom electrode (in contact with the bottom PDMS surface) by a large distance, obscuring the charge-potential relation. Therefore, in our previous work, we directly measured the surface charge density using EFM, which provides an output that can be directly related to the charge density [12]. It produced a reasonable charge density value, but without the KPFM result, we could not validate it through a cross-check [22,23,24,25,26]. EFM is also slower than KPFM. All these lead to the need for enabling KPFM for thick-substrate samples.

To adopt KPFM for the characterization of surface charges on thick-substrate samples, we took the following steps. First, we incorporated the entire KPFM setup, including the thick substrate, into a computational model and numerically solved for the electrostatic potential and fields using a finite difference (FD) electrostatic solver. From the results, we could accurately estimate what fraction of the electrostatic potential difference between the KPFM tip and the ground point is inside the substrate and, hence, to be disregarded from the surface charge density estimation. Second, we adopted the theoretical framework established by Rahe [23] and estimated the surface charge density from the KPFM measurement results. Finally, we performed EFM on the same site and compared the results. We observed order-of-magnitude agreements between the KPFM/electrostatic modeling and EFM results, validating both methods. The details are described below.

**Figure 5:**
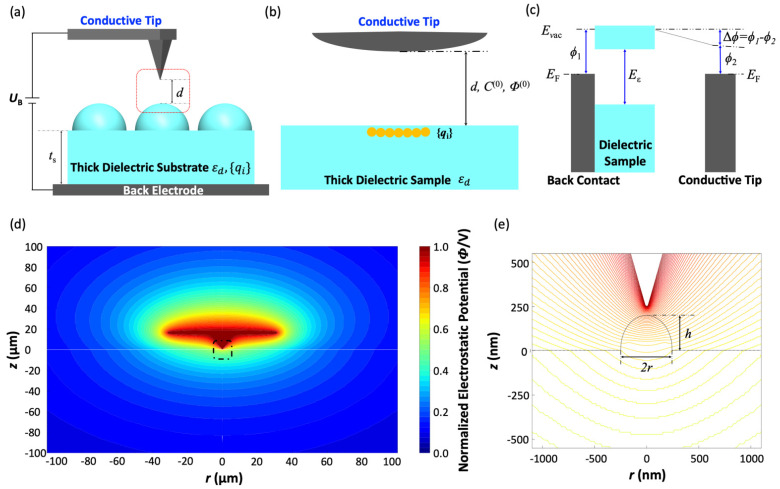
(**a**) The KPFM setup consists of a conductive tip and a dielectric sample ($\varepsilon_{d}$), and the surface charge {*q*_i_} (*U*_B_: sample back contact potential, *t*_s_: sample thickness). (**b**) A magnified view of the tip-sample system (red dashed box in (**a**)). *C*^(0)^ and $\Phi^{\left(0\right)}$ indicate the void tip-sample capacitance and electrostatic potential, respectively. (**c**) Energy diagram of the void tip-sample capacitor. Δϕ is the contact potential difference between the back contact of the sample and the tip. *E*_vac_ and *E*_ε_ represent the vacuum energy level and bandgap of the dielectric sample, respectively. (**d**) The computed electrostatic potential with the tip-sample distance *d* = 50 nm. (**e**) A magnified view of the normalized electrostatic potential distribution (*r* ~ 250 nm, *h* ~ 200 nm), showing the region of the black dashed box in (**d**).

As the FD electrostatic solver, we chose to use CAPSOL, which solves for the electrostatic potential, field, and capacitance in axis-symmetric geometries [22,23,24]. Figure 5a shows the whole KPFM setup with a conductive tip and thick dielectric substrate. The conducting probe has a conical taper terminated by a spherical tip. All dimensions were adopted from the specification of the probe adopted for our experiment. The sample was modeled as a dielectric nanodome with *ε*_d_ = 2.8 (PDMS), and the thickness of sample *t*_s_ was set to 1 mm, which is close to the experimental condition. Computations were performed over various tip-sample distances ranging from *d* = 10 to 100 nm, with an increment ∆*d* = 0.5 nm. The CAPSOL computation was performed with the GNU-GPL licensed Fortran environment. The suggested number of grids points by the CAPSOL code was chosen for the computations. The computations took 15 min on a personal laptop with Intel Dual Core i5 (8th Gen) 1.6 GHz and 8 GB RAM. The resulting electrostatic potential profile in Figure 5d represents the entire potential map with the tip-sample distance, *d*, set to 50 nm to match our experimental setup. Figure 5e shows a magnified view of the electrostatic equipotential lines near the tip and a PDMS nanodome (black dashed box in Figure 5d).

**Figure 6:**
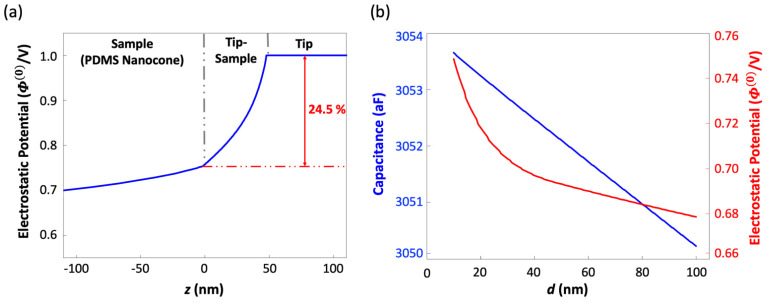
(**a**) The normalized electrostatic potential along the center axis. It shows that 75.5% of total potential drop occurs in the PDMS substrate, leaving only 24.5% of the potential drop to be resulting from the tip-sample surface interaction. (**b**) The electrostatic potential, $\Phi^{\left(0\right)}$, and the void capacitance, *C*^(0)^, extracted from the simulation results as functions of the tip-sample distance, *d*.

From the CAPSOL output, we extracted the electrostatic potential data along the center axis and plotted it in Figure 6a as a function of *z* to find the tip-sample fraction of the electrostatic potential. The plot shows that 75.5% of the potential drop occurs within the dielectric PDMS substrate, leaving only 24.5% of the potential drop occurring between the tip and the sample surface. It implies that the same fraction of the KPFM-measured potential accounts for the tip-surface charge interaction.

With the fractional tip-sample potential drop figured out, we proceeded to apply Rahe’s theoretical framework, which relates the KPFM’s setup and output readings to the surface charge density. In the KPFM setup shown in Figure 5a,b, the point charges {*q_i_*} on the surface interact with the tip and result in a KPFM output potential, *U*_B_, given by:

(1) $$\Delta V_{CPD}=U_{B}=\frac{\Delta \phi }{e}+\frac{\sum_{i}q_{i}\;\frac{\partial }{\partial d}\;\Phi^{\left(0\right)}\left({\vec{r}}_{i}\right)}{\frac{\partial C^{\left(0\right)}}{\partial d}}$$

where Δϕ is the work function difference shown in Figure 5c, *e* is the unit charge, $C^{\left(0\right)}$ is the void capacitance of the setup, $\Phi^{\left(0\right)}\left({\vec{r}}_{i}\right)$ is the normalized void electric potential at ${\vec{r}}_{i}$, and ${\vec{r}}_{i}$ is the position of the point charge, $q_{i}$. The void capacitance, $C^{\left(0\right)}$, and electrostatic potential, $\Phi^{\left(0\right)}\left({\vec{r}}_{i}\right),$ at the surface of the PDMS nanodome can be computed by CAPSOL. By post-processing the result, their partial derivatives with respect to *d*, which is needed in the estimation of charge density, can also be obtained. Since all other parameters in Equation (1) are fixed, *U*_B_ can be directly related to $q_{i}$. For the sake of simplicity, we modeled the surface charge on the PDMS nanodome as a single point charge. To estimate the charge density below the tip on the surface of the PDMS nanodome, we assumed that the electrostatic coupling between the tip and surface occurred over an area of 10^4^ nm^2^ [12]. From the KPFM result and the post-processed CAPSOL computation, the charge density, $\rho_{s,\mathrm{KPFM}}$, in the LE, TE, and IS regions was estimated to be 1.17 × 10^−3^, 2.18 × 10^−4^, and 8.72 × 10^−4^ C/m^2^, or 0.105, 0.014, and 0.055 elementary charges per 10 nm^2^, respectively (Table 1).

To corroborate the resulting KPFM/CAPSOL-based surface charge density estimation, we adopted EFM, which can directly measure the polarity and surface charge density on insulating materials [27,28,29]. In typical EFM setups, the resonance frequency shift, ∆*f*_0_, is related to the force gradient, as [11,12,28,29]:

(2) $$\frac{\Delta f_{0}}{f_{0}}≅-\frac{1}{2k_{c}}\nabla F_{\mathrm{dc}}=-\frac{1}{2k_{c}}\frac{\partial F_{\mathrm{dc}}}{\partial d},$$

where *F*_dc_ is the force exerted on the probe and *k*_c_ is the spring constant of the probe’s cantilever. The right-hand side of Equation (1) can be further expanded to:

(3) $$\Delta f_{o}=-\frac{f_{o}}{2k_{c}}\left(\frac{C^{\prime \prime }}{2}\cdot V_{\mathrm{dc}}^{2}-\frac{q_{s}}{4\pi \varepsilon_{0}}\left(\frac{2C}{d^{3}}-\frac{C^{\prime }}{d^{2}}\right)\cdot V_{\mathrm{dc}}-\frac{q_{s}^{2}}{2\pi \varepsilon_{0}d^{3}}\right)$$

where *C* is the capacitance between the tip and the sample surface, *q*_s_ the surface charge, and *V*_dc_ the dc voltage applied to the tip. It is clear that Equation (3) relates ∆*f*_0_ to *V*_dc_ quadratically. From Equation (3), we can obtain the inflection point:

(4) $$V_{\mathrm{dc}}^{*}=\frac{q_{s}}{4\pi \varepsilon_{0}}\frac{1}{C^{\prime \prime }}\left(\frac{2C}{d^{3}}-\frac{C^{\prime }}{d^{2}}\right)$$

which directly reveals the polarity of *q*_s_ because the other quantities in Equation (4) are always positive [11,12]. By setting *V*_dc_ = 0 V, we could also relate the absolute value of *q*_s_ to ∆*f*_0_ as:

(5) $$\left|q_{s}\right|=\sqrt{\frac{4\pi \varepsilon_{0}\;k_{c}\;d^{3}\;\left|\Delta f_{0}\left(V_{\mathrm{dc}}=0\right)\right|}{f_{0}}}.$$

**Figure 7:**
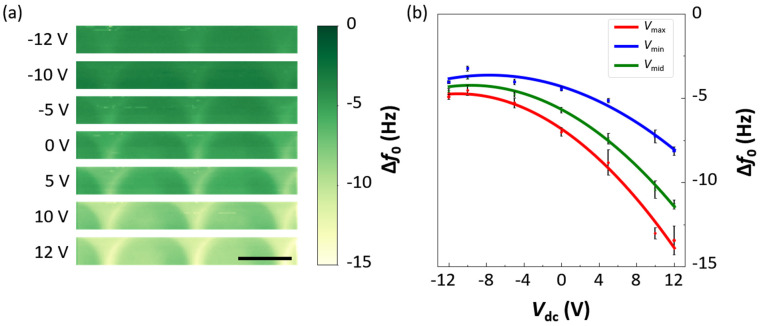
(**a**) EFM images of the PDMS nanodome replica at different values of *V*_dc_ (scale bars: 500 nm). (**b**) The red, blue, and green solid lines represent the resonance frequency shift, Δ*f*_0_, extracted from the LE, TE, and IS regions of the PDMS nanodome in (**a**) as a function of *V*_dc_, respectively. The error bars represent the standard deviation obtained from 2 samples.

For quantitative comparison with the KPFM result, we extracted the resonance frequency shifts, ∆*f*_0_, from Figure 7a and plotted them in Figure 7b as a function of *V*_dc_. As shown in Figure 7b, the blue, green, and red solid lines represent the frequency shifts of the LE, TE, and IS regions, which correspond to the maximum, minimum, and mid-surface charge density regions, respectively. They all exhibit negative parabolic curves, which indicates the existence of negative charges [11,12]. The frequency shifts in the LE, TE, and IS regions at zero bias (*V*_dc_ = 0 V) were measured to be −7, −4.44, and −5.69 Hz, respectively. From the result, the corresponding surface charge density, $\rho_{s,\mathrm{EFM}},\;$values in the LE, TE, and IS regions are 5.39 × 10^−4^, 4.29 × 10^−4^, and 4.86 × 10^−4^ C/m^2^, or 0.0337, 0.0268, and 0.0304 elementary charges per 10 nm^2^, which all agree with the KPFM-based estimation result within a factor of 0.5~3.1, respectively (Table 1). Order-of-magnitude agreements were observed, corroborating the validity of KPFM-based measurements and the surface charge estimation. Both methods showed that the highest charge density will be found in the LE region, although the simulation suggests a larger variation of the surface charge density across the nanodome profile than the EFM measurement.

## 3. Conclusions

In this work, we have provided experimental and theoretical validations to the mechano-triboelectric charging model, which we proposed in our previous work to explain the formation and nanoscale patterning of tribocharges, using multi-physical approaches.

On the experimental side, we confirmed that the mechano-triboelectric charging model can successfully explain the nanopatterned tribocharge formation by replica molding of recessed nanotextures. The spatial distribution patterns of the tribocharge’s surface potential, obtained by KPFM, and the cumulative friction, obtained through computational modeling, agreed well with each other. The leading edge of the nanodome, which underwent the strongest cumulative friction, exhibited the highest surface potential and tribocharge density. Conversely, areas which suffered weak cumulative friction, such as the trailing edge, exhibited low surface potential and tribocharge density. The agreement not only complements the existing mechano-triboelectric charging model but also completes it by confirming its validity for replications of master molds with both recessed and protruded nanotextures.

On the theory side, we refined the KPFM technique for the replica molding-based tribocharging process by integrating it with numerical electrostatic modeling, enabled by CAPSOL. The combined experimental/numerical approach greatly improved the accuracy of the KPFM-based charge density estimation on thick insulating substrates by eliminating the obscurity induced by the existence of the thick dielectric substrate. Specifically, for our setup, the results of the numerical modeling revealed that only 24.5% of the measured potential drop occurred between the conductive tip and the sample surface, prompting us to scale the estimated charge density by the same fraction. We cross-checked the result of the KPFM/CAPSOL approach against the result obtained directly from EFM. The results showed good agreement within factors of 0.5~3.1.

This multi-physical investigation of the replica molding-based tribocharge nanopatterning process will not only broaden our understanding of the nanoscale tribocharge generation but also provide useful and important tools for its study and analysis. We anticipate that the results reported in this paper will advance the technology of nanopatterned charge generation, which can facilitate future energy harvesting and flexible electronics applications.

## Figures and Tables

**Table 1:** Experimental and numerical estimation of the magnitude of the surface charge density on the tribocharged PDMS nanodome at each region (LE, TE, and IS) based on the measurements by EFM and KPFM/CAPSOL.

	Leading Edge (LE)	Trailing Edge (TE)	Interstitial (IS) Region
EFM	0.0337 ^1^	0.0268	0.0304
KPFM/CAPSOL	0.1045	0.0136	0.0545
Ratio	3.101	0.507	1.793

^1^ Unit: elementary charges/10 nm^2^.

## Data Availability

The data presented in this study are available on request from the corresponding author.

## Supplementary Materials

The following are available online at https://www.mdpi.com/article/10.3390/mi12121460/s1, Figure S1: The FEA results of demolding a pyramidal nanostructure.
