# Supplementary material for: Mechano-Triboelectric Analysis of Surface Charge Generation on Replica-Molded Elastomeric Nanodomes
Source: Micromachines (Basel). 2021 Nov 27;12(12):1460. doi: 10.3390/mi12121460 (PMC8705332; doi:10.3390/mi12121460)
Supplement: Supplementary file 1 [file micromachines-12-01460-s001.zip › micromachines-1477897-supplementary.pdf]

Supplementary Information

Mechano-triboelectric Analysis of Surface Charge Generation on Replica-molded Elastomeric Nanodomes

Myung Gi Ji [1,2], Mohammed Bazroun [3], In Ho Cho [3], W. Dennis Slafer [4], Rana Biswas [1,2,5,6,*] and Jaeyoun Kim [1,2,*]

[1] Department of Electrical and Computer Engineering, Iowa State University, Ames, IA 50011, USA; mji90@iastate.edu (M.G.J.)
[2] Microelectronics Research Center, Iowa State University, Ames, IA 50011, USA
[3] Department of Civil, Construction, and Environmental Engineering, Ames, IA 50011, USA; mbazroun@iastate.edu (M.B.); icho@iastate.edu (I.C.)
[4] MicroContinuum Inc., 200 Dexter Avenue, Suite 180, Watertown, MA 02472, USA; (W.D.S.)
[5] Department of Physics and Astronomy, Iowa State University, Ames, IA 50011, USA
[6] Ames Laboratory, Iowa State University, Ames, IA 50011, USA
* Correspondence: biswasr@iastate.edu (R.B.); plasmon@iastate.edu (J.K.)

Supplementary Note 1

To further validate the mechano-triboelectric model obtained from the nanodome shape, we have investigated another nanostructure with a different shape. We chose a pyramidal nanostructure (Fig. S1(a)). Since synthesis of nanostructured molds of arbitrary shape is inherently difficult, we adopted a computational approach. We first simulated the demolding action of a pyramidal shape with aspect ratio ~ h/r=0.4 to compute the lateral sliding distance and frictional stress. After the completion of the demolding action, a nearly linear region of high lateral sliding distance is developed at the trailing edge of the pyramidal structure (Fig. S1(b)). This correlates well with the large frictional stresses shown in Fig. S1(c) that is also developed at the trailing edge of the pyramid. This indicates that the *protruded* nanodomes in Fig. 4(b) and this *recessed* pyramid exhibit qualitatively opposite patterns of lateral sliding distances during the peel-off process. The *protruded* nanodome shows the minimum sliding distance on the trailing edge, whereas the *recessed* pyramid exhibits the maximum sliding distance on it. On the leading edge, the results become reversed. These observations suggest that the mechano-triboelectric model shows good agreements with the expectation and can be applied to a wide variety of mold shapes. In addition, the results obtained from the recessed pyramids are consistent with the measurement and FEA results obtained previously from nanocup molds [1].

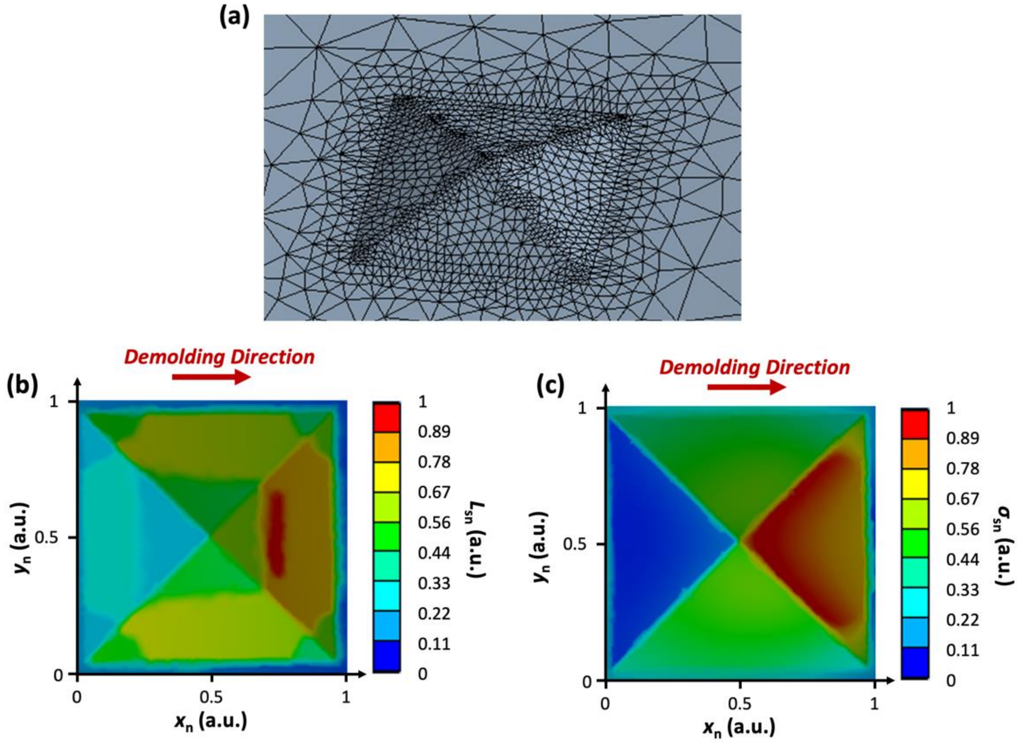

Figure S1. The FEA results of a pyramid structure with aspect ratio h/r ~ 0.4. Due to the length-scale limitation of the continuum FEA in ANSYS, the simulations were done at micrometer length-scales. (a) The setup for FEA of the demolding action of the pyramid structure (b) The top-view of the distribution of the computed lateral sliding distance L_{sn}. (c) The corresponding distribution of frictional stress σ_{sn}. Both the lateral sliding distance and frictional stress are normalized to their maximum values to facilitate the comparison. The demolding directions are set to be left to right and expressed as a red arrow in (b) and (c).

Reference

1. Li, Q.; Cho, I.H.; Biswas, R.; Kim, J. Nanoscale Modulation of Friction and Triboelectrification via Surface Nanotexturing. *Nano Lett.* **2019**, *19*, 850–856, doi:10.1021/acs.nanolett.8b04038.
